# Comparison of the effects of dexmedetomidine and propofol on the cardiovascular autonomic nervous system during spinal anesthesia: preliminary randomized controlled observational study

**DOI:** 10.1007/s10877-023-01062-w

**Published:** 2023-08-12

**Authors:** Han Bum Joe, Yun Jeong Chae, Seung Ho Song, In Kyong Yi

**Affiliations:** Department of Anesthesiology and Pain Medicine, Ajou University School of Medicine, 164, World cup- ro, Yeongtong-gu, Suwon, 16499 Republic of Korea

**Keywords:** Autonomic nervous system, Dexmedetomidine, Hemodynamic, Moderate sedation, Propofol, Spinal anesthesia

## Abstract

Spinal anesthesia induces sympatholysis and is usually combined with dexmedetomidine or propofol which induce different hemodynamic changes. The purpose of this study was to compare the effect on autonomic nervous system between dexmedetomidine and propofol combined with spinal anesthesia. Patients aged 20–65 undergoing elective surgery under spinal anesthesia were randomly assigned to dexmedetomidine or propofol group. Heart rate variability (HRV) and hemodynamic variables were measured at four time points: T0, baseline; T1, 10 min after spinal anesthesia; T2, 10 min after sedative administration; and T3, 20 min after sedative administration. In 59 patients, dexmedetomidine and propofol groups had significantly different hemodynamic changes over time (time × group effect P < 0.001). The dexmedetomidine group had slower heart rate at T2 (P = 0.001) and higher blood pressures at T2 and T3 (P < 0.001) than the propofol group. Overall HRV dynamics showed a significant change over time from T0 to T3, but both groups exhibited similar trends. Compared to the baseline data within the group, the low frequency (LF) decreased in both groups but the decrease occurred at T2 in the propofol group and at T3 in the dexmedetomidine group. The high frequency (HF) increased at T2 and T3 only in the dexmedetomidine group. The LF/HF ratio decreased in the dexmedetomidine group at T3. Dexmedetomidine showed slower heart rate and higher blood pressure than propofol when combined with spinal anesthesia, however, dexmedetomidine and propofol exhibited similar trends in HRV dynamics. Compared with the baseline within each group, both agents decreased LF, but only dexmedetomidine increased HF and decreased in the LF/HF ratio significantly.

## Introduction

Spinal anesthesia causes iatrogenic central sympatholysis by blocking the pre-ganglionic sympathetic fibers and cardiac sympathetic innervation. This iatrogenic sympathetic blockage causes hypotension and bradycardia, which occasionally require clinical intervention [1]. Heart rate variability (HRV) is widely used as a standard method to assess the autonomic nervous system [2]. The HRV signal is derived from the RR interval of the EKG, originates from the brain, and is mediated through the sympathetic and parasympathetic nervous systems that innervate the sinoatrial node [3]. A previous study showed that the total power and low frequency (LF) / high frequency (HF) ratio, among the parameters of HRV, decreased after spinal anesthesia compared with before spinal anesthesia [4]. A decrease in total power reflects a decrease in overall autonomic nervous activity, and a decrease in the LF/HF ratio reflects a relative decrease in sympathetic activity compared to parasympathetic activity [5].

In clinical practice, spinal anesthesia is usually combined with sedation, mainly using propofol or dexmedetomidine. In addition to spinal anesthesia, propofol and dexmedetomidine induce hemodynamic changes and alter the autonomic nervous system. Previous studies have reported that propofol induces bradycardia and hypotension [6] and decreases the overall HRV [7–9]. Dexmedetomidine, a highly selective α_2_ agonist, causes hemodynamic changes different than those caused by propofol, including transient hypertension, profound bradycardia, and hypotension due to pre and postsynaptic α_2_ receptor activation [10]. Previous studies have reported mixed results of the effect of dexmedetomidine on HRV. Hogue et al. [11] reported that dexmedetomidine reduced LF power, but had little or no effect on HF power, whereas Tarvainen et al. [12] reported that dexmedetomidine increased HF power. In dexmedetomidine combined with spinal anesthesia, one study showed that the total power and LF/HF ratio decreased compared to pre-spinal anesthesia [13]. In propofol combined with spinal anesthesia, a previous study showed that LF power and LF/HF ratio decreased [14]. However, no studies have directly compared the effects of dexmedetomidine and propofol on the HRV dynamics when combined with spinal anesthesia.

Spinal anesthesia is known to reduce sympathetic activity, and sometimes requires clinical intervention. Since dexmedetomidine and propofol have been shown to induce different hemodynamic changes, we hypothesized that they would also cause different changes in the autonomic nervous system additional to the effect of spinal anesthesia. Such information could be valuable in selecting a sedative for use in spinal anesthesia. The present preliminary study aimed to compare the differences in HRV dynamic during sedation with propofol or dexmedetomidine in patients who underwent spinal anesthesia.

## Materials and methods

This study was approved by the Ajou Institutional Review Board on October 29, 2019 (AJIRB-MED-INT-19-350) and the Clinical Trial registration was done prior to the enrollment of the first patient (www.clinicaltrials.gov, NCT04142502). Written informed consent was obtained from all the participants. This prospective randomized preliminary study enrolled patients aged 20–65 years with American Society of Anesthesiologists physical status classifications I–III, who were scheduled for elective surgery under spinal anesthesia. The exclusion criteria were patients with diabetes mellitus, arrhythmias of any kind, thyroid hormone disorders, or those taking medications that could affect the autonomic nervous system (i.e., psychiatric medication and beta-blockers). Patients who were unable to cooperate with HRV measurements for 3 min were also excluded. Y. J. C randomly assigned eligible patients to the dexmedetomidine or propofol groups using a computer-generated random numbers table. S. H. S. recorded the HRV data, and H. B. J. performed spinal anesthesia and sedation.

### Measurement of HRV

HRV was measured using an SA-3000P (Medicore Co., Ltd. Hanam, Gyeonggi-Do, Korea). Baseline HRV was measured on the morning of the day before surgery in a quiet room in the ward. The patient rested for 5 min before the measurement, and electrocardiogram (EKG) leads were applied. During the measurement, conversation was prohibited, and the patients were asked to open their eyes and stare at an empty wall in a supine position. Subsequent to the baseline measurement, three measurements were performed as follows: T1, 10 min after spinal anesthesia; T2, 10 min after the start of sedative administration; and T3, 20 min after the start of sedative administration. All measurements were conducted by collecting data for at least 3 min and while patients were in a supine position.

The collected HRV parameters were calculated automatically by the SA-3000P as follows:

- Time domain and complexity parameters.

SDNN: standard deviation of the NN interval

RMS-SD: square root of the mean of the sum of the squares of the differences between adjacent NN interval

PSI: physical stress index or pressure index

ApEn: approximate entropy

SRD: successive RR interval difference

- Frequency domain parameters.

Total power

VLF: very low frequency, 0.003–0.004 Hz

LF: low frequency, 0.04–0.15 Hz

HF: high frequency, 0.15–0.4 Hz

LF Norm (n.u.): Normalized LF = LF / LF + HF

HF Norm (n.u.): Normalized HF = LF / LF + HF

LF/HF ratio: low frequency / high frequency

### Anesthesia

Patients entered the operating room with no premedication. Standard monitoring of pulse oximetry, EKG, noninvasive blood pressure (BP), and bispectral index (BIS, Medtronic, Minneapolis, MN, USA) was performed. After measuring baseline hemodynamics, 5 L/min of oxygen was administered using a facial mask. The patient was placed in a lateral position with the operating leg facing down, and spinal anesthesia was administered at the lumbar 3/4 or 4/5 level with a 25G spinal needle. Hyperbaric bupivacaine (0.5%) was administered differentially according to the patient’s height with the aim of achieving an anesthesia level of T 10. Ten minutes after the induction of spinal anesthesia, the anesthesia level was checked using an alcohol swab. Hemodynamic parameters and T1 HRV were then measured.

Thereafter, dexmedetomidine or propofol sedation was initiated according to group assignment. In the dexmedetomidine group, 0.6 mcg/kg was loaded for the first 10 min, followed by infusion at 0.5 mcg/kg/h. In the propofol group, the effect-site concentration of 0.3–1.0 ng/mL was adjusted using a target concentration infusion to maintain the BIS at 60–80. In both groups, T2 and T3 HRV were measured at 10 and 20 min, respectively, from the initial sedative administration. In cases of bradycardia (heart rate < 50 beats/min), atropine (0.5 mg) was administered, and ephedrine (8 mg) was administered for hypotension (systolic blood pressure < 90 mmHg).

### Statistical analysis

All statistical analyses were performed using SPSS (version 26.0; IBM Corp., Armonk, NY, USA). For continuous data, normality was assessed using the Shapiro–Wilk test, and the independent *t* test or Mann–Whitney U test was performed for normally or non-normally distributed data, respectively. Chi-squared or Fisher’s exact tests were used to compare categorical data between the two groups. Repeated measures data were analyzed using generalized estimating equations for non-parametric data and repeated measures ANOVA for parametric data. For repeated measurements, the Bonferroni post-hoc test was performed. Data were described as mean ± standard deviation (SD), median and interquartile range (IQR), or as a proportion of patients (%). P *<* 0.05 was considered statistically significant.

## Results

### Demography

**Fig. 1:**
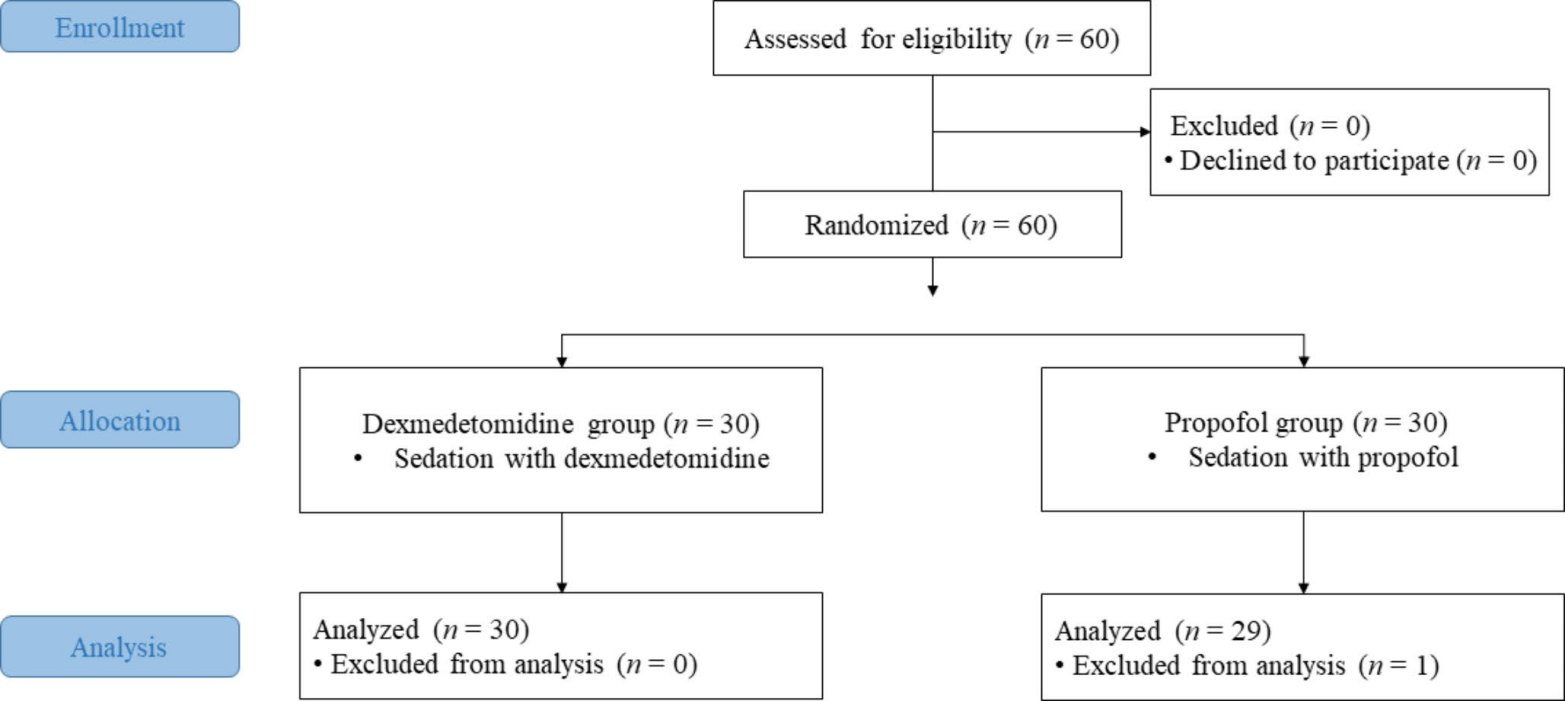
Flow diagram

Sixty patients completed the study (Fig. 1). One patient’s HRV data was not stored properly; therefore, data from one patient in the propofol group were excluded from the analysis. Demographic data, spinal anesthesia level, and the number of vasoactive drugs used did not differ between the two groups (Table 1).

**Table 1:** Patient characteristics and intraoperative data

	D group (n = 30)	P group (n = 29)	P value
Age, years	58 ± 3	58 ± 4	0.867
Height, cm	160 ± 9	157 ± 7	0.137
Weight, kg	66.5 ± 9	67 ± 11	0.794
Male:Female	10:20	4:25	0.078
Spinal anesthesia level			
T7 / T8 / T9 / T10 / T11	1 / 8 / 6 / 15 / 0	1 / 7 / 8 / 12 / 1	0.878
Vasoactive drug	8 (26.7)	7 (24.1)	1.000
Atropine	8 (26.7)	3 (10.3)	0.181
Ephedrine	1 (2.5)	4 (13.8)	0.195

Values are mean ± standard deviation and number (%)

D group, dexmedetomidine group; P group, propofol group

### Dexmedetomidine group vs. propofol group

**Fig. 2:**
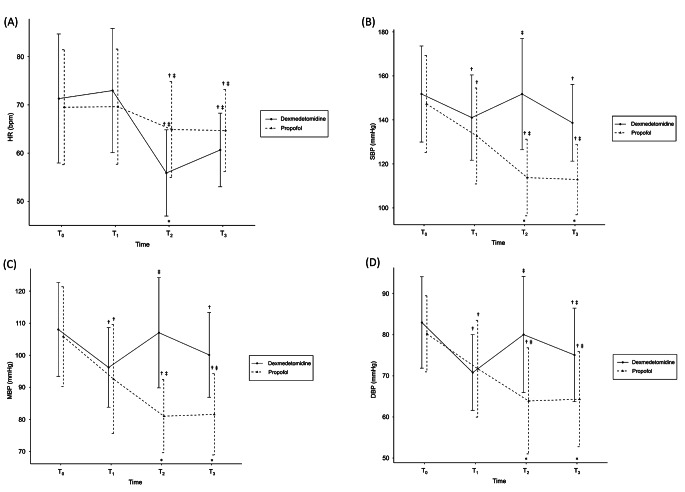
Hemodynamic change **(A)** Heart rate, **(B)** Systolic blood pressure, **(C)** Mean blood pressure, **(D)** Diastolic blood pressure Values are presented as the mean ± standard deviation. T0, baseline; T1, 10 min after spinal anesthesia; T2, 10 min after start of sedative administration; T3, 20 min after start of sedative administration. * P **<** 0.05/4, dexmedetomidine vs. propofol; † P < 0.05/3, compared with T0 within group; ‡ P < 0.05/3, compared with T1 within group

Hemodynamic data, including heart rate (HR), systolic blood pressure (SBP), mean blood pressure (MBP), and diastolic blood pressure (DBP) showed significantly different trends between the two groups. The p-values of the time effect and time × group effect were all < 0.001 (Fig. 2). In both groups, the HR decreased compared with that at T0, but the decrease was greater in the dexmedetomidine group. The difference between the two groups was most pronounced in T2 (56 ± 9 vs. 65 ± 10 bpm, p = 0.001). In the propofol group, BP decreased at T2 and T3 compared to that at T0 and T1. In contrast, in the dexmedetomidine group, BP decreased after spinal anesthesia (T1), returned to the baseline level at T2, then decreased again at T3. Therefore, the difference between the two groups was pronounced at T2. At T2: SBP, 152 ± 25 vs. 114 ± 17 mmHg, P < 0.001; MBP, 107 ± 17 vs. 81 ± 11 mmHg, P < 0.001; and DBP, 80 ± 14 vs. 64 ± 13 mmHg, P < 0.001for the dexmedetomidine vs. propofol groups, respectively. At T3, the dexmedetomidine group showed a higher BP than the propofol group. At T3: SBP, 139 ± 18 vs. 113 ± 16 mmHg, P < 0.001; MBP, 100 ± 13 vs. 82 ± 13 mmHg, P < 0.001; and DBP, 75 ± 11 vs. 64 ± 12 mmHg, P = 0.001. The level of BIS was lower in the dexmedetomidine group than in the propofol group at T3, despite a higher BP (61 ± 8 vs. 72 ± 7, P < 0.001).

**Fig. 3:**
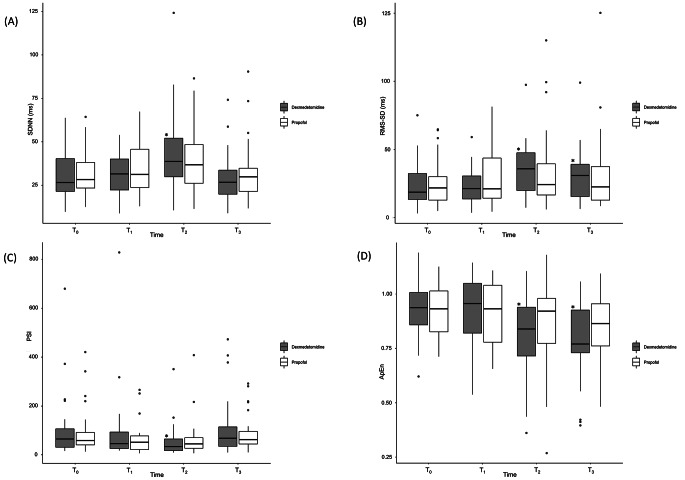
Time domain and complexity parameters **(A)** SDNN, **(B)** RMS-SD, **(C)** PSI, **(D)** ApEn. All box-and-whisker plots represent values within the interquartile range (IQR, boxes), median (horizontal lines inside the boxes) and 1.5 × IQR (whiskers). Outliers (solid dots) are plotted as values > 1.5× IQR. * P **<** 0.05/3 compared to T0 value in intra-group analysis SDNN,standard deviation of the NN interval; RMS-SD, square root of the mean of the sum of the squares of the differences between adjacent NN interval; PSI, physical stress index or pressure index; ApEn, approximate entropy T0, baseline; T1, 10 min after spinal anesthesia; T2, 10 min after start of sedative administration; T3, 20 min after start of sedative administration

Overall HRV dynamics showed a significant change over time from T0 to T3, but both groups exhibited similar trends. The time-domain parameters are presented in Fig. 3. Before showing other results, the SRD, which examines whether the data maintained in a constant status, showed values close to 1 and no significant difference between the two groups. (P = 0.063). All time-domain and complexity parameters, including SDNN, RMS-SD, PSI, and ApEn, showed similar trends in both groups. While the time effect was significant (SDNN, P < 0.001; RMS-SD, P = 0.010; PSI, P = 0.001; and ApEn, P < 0.001), no significant group or time × group effects were observed. This indicates that the time-domain parameters were changed by sedative administration, but there was no difference between the two groups. In the dexmedetomidine group, SDNN increased at T2 (T0→T2, 27 [21–41] vs. 39 [28–53] ms, P = 0.004) and RMS-SD increased at T2 and T3 (T0→T2, 19 [13–33] vs. 36 [18–48] ms, P < 0.001; and T0→T3, 19 [13–33] vs. 31 [15–41] ms, P = 0.002). PSI increased at T2 (T0→T2, 65 [30–112] vs. 34.126 [18–67], P = 0.006) and ApEn decreased at T2 and T3 compared to that at T0 (T0→T2, 0.94 [0.85–1.01] vs. 0.84 [0.70–0.94], P = 0.002; T0→T3, 0.94 [0.85–1.01] vs. 0.77 [0.72–0.93], P = 0.001). In the propofol group, none of the parameters were significantly different from those at T0.

**Fig. 4:**
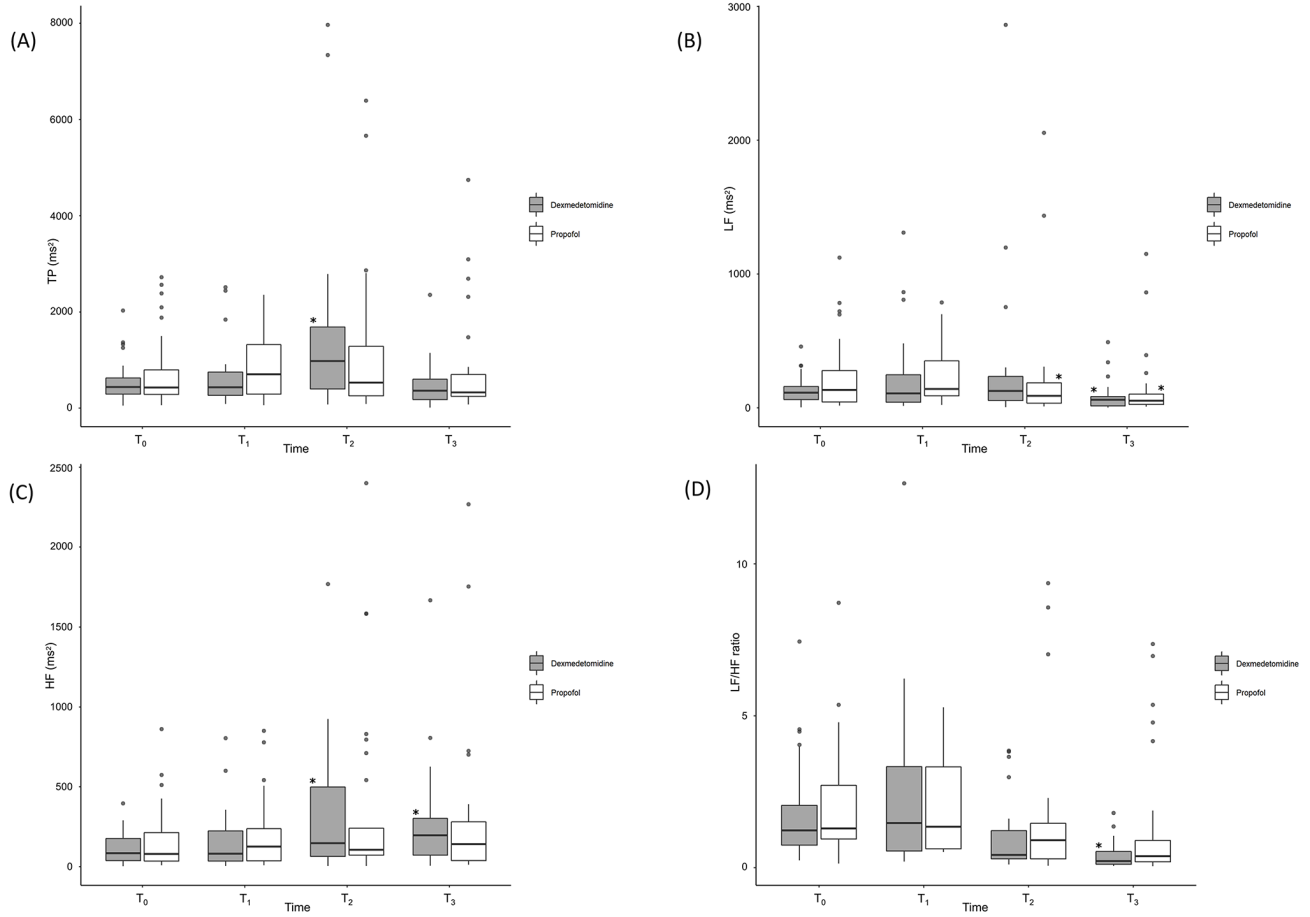
Frequency domain parameters **(A)** TP, **(B)** LF, **(C)** HF, **(D)** LF/HF ratio All box-and-whisker plots represent values within the interquartile range (IQR, boxes), median (horizontal lines inside the boxes) and 1.5 × IQR (whiskers). Outliers (solid dots) are plotted as values > 1.5× IQR. * P **<** 0.05/3 compared to T0 value in intra-group analysis TP, total power; LF, low frequency; HF, high frequency T0, baseline; T1, 10 min after spinal anesthesia; T2, 10 min after start of sedative administration; T3, 20 min after start of sedative administration

The frequency domain parameters are presented in Fig. 4. All parameters changed with time in a similar manner in both groups (time effect: total power, P = 0.009; LF, P < 0.001; HF, P = 0.005; LF Norm, P < 0.001; HF Norm, P < 0.001; and LF/HF ratio, P < 0.001). However, no significant difference between the two groups were observed because the group effect and time × group effect were not statistically significant for any of the frequency domain parameters. However, there were some differences in the patterns of change in intra-group analysis. The total power doubled at T2 compared with that at T0 only in the dexmedetomidine group (414 [269–681] vs. 904 [369–1731] ms^2^, P = 0.001), which was not observed in the propofol group. The LF showed a tendency to decrease in both groups, but the decrease occurred at different times. In the propofol groups, it decreased after T2 (P = 0.008), whereas it decreased later in the dexmedetomidine group (T0→T3; P = 0.015). HF increased at T2 and T3 compared to that at T0 only in the dexmedetomidine group (T0→T2, P = 0.001; T0→T3, P = 0.002). The LF/HF ratio decreased at T3 compared to that at T0 only in the dexmedetomidine group (T0→T3, P < 0.001).

### High baseline LF/HF ratio vs. low baseline LF/HF ratio

A previous study reported that an preoperative LF/HF ratio > 2.3 was correlated with post-spinal anesthesia hypotension [15]. Based on this report, we divided patients into high LF/HF (> 2.3) and low LF/HF (≤ 2.3) groups and performed secondary analysis in all patients and subgroup analysis in the dexmedetomidine group and the propofol group, respectively. Primary analysis of this study confirmed that dexmedetomidine and propofol induced different changes in hemodynamics. Therefore, the prediction of hypotension based on the LF/HF ratio could be influenced by the sedative used.

### All patients

Of the 59 patients, 45 were in the low LF/HF ratio group and 14 were in the high LF/HF ratio group. Vasoactive drugs were required in 10 patients (22.2%) in the low LF/HF ratio group and in five patients (35.7%) in the high LF/HF ratio group, and it was not statistically significant (P = 0.483). The hemodynamic data showed no significant differences between the two groups (Table 2).

**Table 2:** Subgroup analysis according to LF/HF ratio: Hemodynamic data

In all subject	Low ratio (n = 45)	High ratio (n = 14)	P value	Group	Time	Time*Group
HR, bpm				0.409	0.000	0.584
T0	68 [60–76]	77 [66–82]	0.203			
T1	72 [61–79]	70 [63–80]	0.843			
T2	58 [53–67]	60 [54–68]	0.675			
T3	61 [56–68]	63 [60–65]	0.346			
SBP, mmHg				0.652	0.000	0.575
T0	152 [134–168]	145 [140–157]	0.399			
T1	135 [126–149]	141 [118–163]	0.569			
T2	130 [111–156]	132 [106–153]	0.741			
T3	121 [109–142]	124 [103–137]	0.569			
MBP, mmHg				0.221	0.000	0.080
T0	106 [98–121]	99 [92–113]	0.069			
T1	94 [84–105]	95 [84–110]	0.606			
T2	94 [80–111]	88 [80–105]	0.351			
T3	89 [81–104]	84 [74–94]	0.179			
DBP, mmHg				0.498	0.000	0.824
T0	82 [76–89]	76 [71–89]	0.197			
T1	72 [63–79]	70 [62–83]	0.933			
T2	72 [61–83]	66 [59–84]	0.445			
T3	70 [61–80]	65 [58–76]	0.404			
In D group	Low ratio (n = 24)	High ratio (n = 6)	P value	Group	Time	Time*Group
HR, bpm				0.614	0.000	0.626
T0	71 [61–77]	68 [61–82]	0.965			
T1	74 [65–80]	68 [63–80]	0.571			
T2	55 [51–62]	54 [46–60]	0.948			
T3	59 [54–67]	62 [59–65]	0.405			
SBP, mmHg				0.757	0.000	0.671
T0	155 [130–169]	152 [140–173]	0.742			
T1	135 [129–152]	149 [118–166]	0.424			
T2	152 [134–168]	143 [132–167]	0.954			
T3	139 [124–148]	135 [130–151]	0.756			
MBP, mmHg				0.666	0.000	0.194
T0	112 [97–121]	108 [93–119]	0.879			
T1	95 [88–105]	93 [83–118]	0.729			
T2	110 [95–116]	101 [88–116]	0.579			
T3	100 [89–108]	92 [88–114]	0.669			
DBP, mmHg				0.552	0.000	0.110
T0	83 [77–88]	85 [68–103]	0.577			
T1	70 [64–79]	67 [61–84]	0.835			
T2	80 [71–91]	74 [61–87]	0.424			
T3	77 [67–84]	66 [60–88]	0.304			
In P group	Low ratio (n = 21)	High ratio (n = 8)	P value	Group	Time	Time*Group
HR, bpm				0.202	0.016	0.329
T0	64 [58–75]	78 [70–83]	0.052			
T1	66 [59–78]	72 [60–84]	0.657			
T2	63 [56–71]	64 [59–79]	0.790			
T3	63 [59–68]	64 [60–72]	0.795			
SBP, mmHg				0.663	0.000	0.361
T0	149 [136–168]	141 [130–148]	0.124			
T1	135 [120–146]	138 [103–163]	0.731			
T2	112 [102–124]	110 [101–143]	0.728			
T3	109 [103–121]	105 [97–122]	0.463			
MBP, mmHg				0.294	0.000	0.076
T0	104 [97–124]	99 [91–101]	0.048			
T1	93 [81–105]	98 [74–108]	0.607			
T2	78 [73–91]	81 [71–87]	0.933			
T3	80 [73–92]	77 [68–82]	0.272			
DBP, mmHg				0.940	0.000	0.456
T0	81 [75–90]	73 [71–78]	0.042			
T1	75[61–79]	73 [61–82]	0.952			
T2	60 [56–71]	64 [51–74]	0.876			
T3	62 [53–76]	62 [56–71]	1.000			

Values are median [interquartile range]

Low ratio, LF/HF ratio ≤ 2.3; high ratio, LF/HF ratio > 2.3

D group, dexmedetomidine group; P group, propofol group

HR, heart rate; SBP, systolic blood pressure; MBP, mean blood pressure; DBP, diastolic blood pressure

T0, baseline; T1, 10 min after spinal anesthesia; T2, 10 min after sedation; T3, 20 min after sedation

### Dexmedetomidine group

Of the patients in the dexmedetomidine group, six were in the high LF/HF ratio group and 24 were in the low LF/HF group. Vasoactive drugs were required in two patients (33.3%) in the high LF/HF ratio group and in six patients (25.0%) in the low LF/HF ratio group. However, this difference was not statistically significant. The hemodynamic data showed no differences between the two groups (Table 2).

### Propofol group

Of the patients in the propofol group, eight were assigned to the high LF/HF ratio group and 21 to the low LF/HF ratio group. Vasoactive drugs were required in three patients (37.5%) in the high LF/HF ratio group and in four patients (19.0%) in the low LF/HF ratio group. Although the difference was not statistically significant, the proportion of patients requiring vasoactive drugs in the high LF/HF group was approximately twice that of those in the low LF/HF group. This difference was larger than that between the low and high LF/HF groups in the dexmedetomidine group. In the hemodynamic data, none of the parameters showed a significant time × group effect (Table 2).

## Discussion

This randomized controlled trial showed that HRV dynamics changed through spinal anesthesia and sedation, however, dexmedetomidine and propofol exhibited similar trends in HRV dynamics. Although dexmedetomidine and propofol did not result any significant difference in the HRV dynamic, the difference in hemodynamic change was obvious between the two groups. Dexmedetomidine induced more bradycardic and less hypotensive in hemodynamic changes compared to propofol. In intragroup analysis compared to each group’s baseline, the two sedatives showed distinctive features. Both sedatives decreased sympathetic activity, but dexmedetomidine did so at a later time, and only dexmedetomidine increased the parasympathetic activity. The LF/HF ratio was decreased only in dexmedetomidine.

Spinal anesthesia has known to cause an iatrogenic sympathetic block, which leads to hypotension and bradycardia, requiring intervention. However, our data showed that spinal anesthesia itself had no effect on HRV dynamics. In this study, HRV data measured at T1 (10 min after spinal anesthesia) could be considered as the pure effect of spinal anesthesia, because it was measured prior to sedative administration, and the HRV data at T1 showed no statistically significant change compared to that at T0. A study by Hidaka et al. [14] corroborates our findings, showing that spinal anesthesia itself had no effect on power spectral changes. In that study, it was thought that the level of anesthesia was not sufficiently high, so it did not seem to affect the HRV dynamics. Further, the level of spinal anesthesia was T6–T11 in Hidaka’s study; however, pre-ganglionic sympathetic cardiac accelerator fibers originate from higher levels such as T1–T4. The level of spinal anesthesia in our patients ranged from T7–T11. Previous studies reported that LF decreases and HF increases; therefore, the LF/HF ratio decreases after spinal anesthesia [16, 17]. However, the level of spinal anesthesia increased to T2–T5 in these studies, which is relatively higher than the results of our study and those of Hidaka. Therefore, it can be concluded from our data that relatively low-level spinal anesthesia did not cause any change in HRV for 10 min after administration.

Although no statistically significant difference was noted in the overall HRV dynamics between dexmedetomidine and propofol, we found variation in the patterns of HRV dynamics. Intragroup analysis of each group showed that LF, which represents sympathetic heart rate modulation, decreased with both sedatives, compared to that at baseline. In contrast, HF, which represents parasympathetic heart rate modulation, increased only with dexmedetomidine, compared to that at baseline (Fig. 4). The parasympathetic activation by dexmedetomidine may be related to its unique hemodynamic changes that induce initial hypertension and bradycardia. Dexmedetomidine is a highly selective α2-adrenoreceptor agonist that also acts on the peripheral α2-adrenoreceptor. Peripheral α2-receptor-mediated arterial vasoconstriction leads to hypertension, and baroreflex-mediated parasympathetic activation is assumed to lead to bradycardia in response to increased BP [18–20]. Some studies have reported that dexmedetomidine increases HF power [12, 20], whereas others have reported that dexmedetomidine decreases LF power, but does not affect HF power [11]. These mixed results show that dexmedetomidine induces a relatively high parasympathetic activity compared to sympathetic activity.

Another hypothesis could explain the increase in HF power with only dexmedetomidine. This may be related to the timing of HRV measurement. Tarvainen et al. [12] evaluated the HRV dynamics during the loss and recovery of consciousness using dexmedetomidine and low-dose propofol. They reported that just prior to loss of consciousness, HF power increased with both drugs. In this study, we did not measure HRV at the time point of loss of consciousness but measured HRV at 10 and 20 min after initiating the sedation drug. No change in HF was induced by propofol in this study, possibly because we could not capture the timing of the loss of consciousness, which occurred within 1 min of propofol administration.

In this study, dexmedetomidine showed favorable hemodynamics compared with propofol in patients who underwent spinal anesthesia. Dexmedetomidine showed a delayed decrease in sympathetic activity compared with propofol (T3 in the dexmedetomidine group and T2 in the propofol group). If sympathetic activity correlates well with BP, it can provide useful information for cardiovascular high-risk patients. By adjusting the initiation timing of sedative administration, it is possible to achieve more favorable results for BP maintenance in high-risk patients under spinal anesthesia.

In the second analysis, we examined whether the LF/HF ratio predicted spinal anesthesia-induced hypotension, as reported in previous studies [21, 22]. In our study, no difference in hemodynamic trends and HRV dynamics between the high and low LF/HF ratio groups were observed for each sedative. It is worth noting that, for propofol, the proportion of patients requiring vasoactive drugs in the high LF/HF ratio group was approximately twice that in the low LF/HF ratio group (19% vs. 37.5%). Although it was not statistically significant due to the small sample size, the possibility of hypotension prediction of the LF/HF ratio can be suggested for spinal anesthesia with propofol sedation. This phenomenon was less pronounced following dexmedetomidine administration.

It is assumed that the prediction of hypotension using the LF/HF ratio cannot be applied to all anesthetics. Most of the anesthetics decreased the sympathetic heart rate modulation, but for the parasympathetic heart rate modulation, the change pattern varied (increased or decreased), and the degree of the decrease differed depending on the agent. Previous studies have shown that various anesthetics induce dynamic changes in HRV. With sevoflurane, LF decreased with a reduction in the BIS value, but HF decreased after induction, and no further decreases were observed despite the reduction in the BIS value [9]. Remimazolam decreased LF and HF but maintained the LF/HF ratio [23]. The increase in the parasympathetic activity of dexmedetomidine has known to be related to peripheral vasoconstriction. If other anesthetics have similar mechanisms of parasympathetic activity, the correlation between the preoperative LF/HF ratio and hypotension may not be sufficiently high. Therefore, it is necessary to determine whether the LF/HF ratio can predict hypotension associated with various drugs. According to our data, it is possible that the LF/HF ratio could predict hypotension with propofol, but this seems less likely with dexmedetomidine.

The current study, as a preliminary observational study, showed how the autonomic nervous system is altered under spinal anesthesia and sedation. The results of this study have the potential to be used as a reference in various fields. For example, a delayed decrease in sympathetic activity induced by dexmedetomidine could help determine the timing of sedation during spinal anesthesia. But at the same time, care must be taken when judging and applying the results from HRV measurement. This study showed that sympathetic activity and BP are not always correlated, as other compound factors such as peripheral vasoconstriction may play a role. Bradycardia is caused not only by an increase in parasympathetic tone but also by sympathetic withdrawal or sympathetic-parasympathetic interaction. HRV detects only information from the heart-brain interactions, interference from other components is not detected. Interpreting LF and HF component of HRV as sympathetic and parasympathetic tone is oversimplified and could lead to a wrong conclusion [2]. Nevertheless, HRV provide the information for circulatory regulation generated by brain. This study offers insights ANS change that occur concomitantly with changes in HR and BP during sedation combined with spinal anesthesia using HRV.

This study had a few limitations. First, the individual variations in HRV values were larger than the sample size. In previous study, the challenges of conducting research due to inter-individual variation have been mentioned [12]. Second, because the hemodynamic data were measured at a specific time point rather than the average of a specific period, there was a limit to the power of the data. Third, as this was a preliminary study, the primary outcomes were unclear.

## Conclusion

Dexmedetomidine showed slower heart rate and higher blood pressure than propofol when combined with spinal anesthesia, however, dexmedetomidine and propofol exhibited similar trends in HRV dynamics. Compared with the baseline within each group, both agents decreased LF, but only dexmedetomidine increased HF and decreased in the LF/HF ratio significantly.
